# The impact of the inclusive financial development index on farmer entrepreneurship

**DOI:** 10.1371/journal.pone.0216466

**Published:** 2019-05-07

**Authors:** Lili Jiang, Aihua Tong, Zhifei Hu, Yifeng Wang

**Affiliations:** 1 Business School, Suqian College, Suqian, JiangSu, China; 2 Shenwan Hongyuan Securities CO., LTD, Shanghai,China; University of Toronto, Rotman School, CANADA

## Abstract

Based on the calculation of the inclusive financial development level of 22 provinces and 4 municipalities in China from 2004 to 2017, this paper uses the Kernel density estimation method to further analyze the evolution of the inclusive financial index. Based on the above analysis, we make empirical analysis of the impact of China's inclusive financial inclusion development index on farmers' entrepreneurship using static panel and dynamic panel estimation method. The empirical conclusions show that there are certain differences in inclusive financial inclusion development level in various provinces in China. Improving the inclusion development level of inclusive finance can better promote farmers' entrepreneurship. Urbanization level, economic openness and regional economic development level have a significant positive effect on farmers' entrepreneurship, while farmers' income and education level have a significant negative effect on farmers' entrepreneurship. It is possible to promote farmers' entrepreneurship by improving the inclusive development level of inclusive finance, combining urbanization, increasing government investment in productive fixed assets, increasing economic openness and improving regional economic development.

## Introduction

Since the “2005 International Year of Microcredit” first proposed inclusive finance, inclusive finance has been widely recognized as a financial development strategy by all countries in the world[1]. In 2016, the State Council of China issued the “Promoting Inclusive Financial Development Plan (2016–2020)”, which proposed the overall goal of China's development of inclusive finance. The essence of inclusive finance is to hope that all groups can enjoy fair and sustainable financial services, especially low-income groups and vulnerable groups with financing difficulties. One of the main problems faced by farmers in the process of entrepreneurship is financing constraints. Under this circumstance, the government can make more emphasis on “blind spots” that farmers have difficulty covering in the past by promoting inclusive finance, which is beneficial to the promotion of farmers' entrepreneurship and is beneficial to the development of rural economy. Therefore, it is particularly important to measure the development level of China's inclusive finance and analyze its impact on the entrepreneurship of farmers, which providing a corresponding basis for the government to formulate specific policies to support farmers' entrepreneurship. What is China's current level of inclusive financial development? What is the impact of inclusive financial development on farmers 'entrepreneurship? This paper make an in-depth analysis on the above two issues.

Inclusive finance can provide appropriate financial services to vulnerable groups in the weak links of financial services such as small and micro enterprises, farmers, low-income groups, poor people and people with disabilities. Inclusive finance is a kind of shared service finance. People with financial needs have equal opportunities, which can change individual economic behaviors. It can play a role in optimizing the allocation of funds and promote economic growth and social progress. There are several definitions of inclusive finance at home and abroad: The United Nations convened a global conference on building an inclusive financial system and proposed the term “inclusive finance” in May 2005. Helms (2006) proposed to change the financial exclusion of the traditional financial system, especially for the majority of customers excluded from the formal financial system to obtain financial services, which can be said to be the direct purpose of developing inclusive finance[2]. Easterly (2006) proposed that inclusive finance has always been regarded as an important mechanism that can solve poverty problems, promote economic growth, and achieve an inclusive society[3]. In addition, Sarma & Pais (2011) pointed out that inclusive finance is relative to financial exclusion, which means that all economic entities can enjoy and use financial products and services at a reasonable price[4]. ^f^Chinese scholars also put forward the corresponding meaning of inclusive finance based on the study of foreign literatures and combined with China's national conditions: Jiao Jinpu (2006) first proposed an inclusive financial system, who believes that inclusive finance is in microfinance and its extension and development. The inclusive financial system is based on the pursuit of sustainability, providing financial systems including savings, credit, insurance and other financial services to all sectors, including vulnerable groups excluded from the traditional financial system[5]. Du Xiaoshan (2006) proposed that the inclusive financial system is a financial system that integrates micro-inclusive financial services objects, meso-inclusive financial providers and macro financial systems and the services of inclusive financial systems should include the poor and low-income groups particularly[6].^.^

Economists have conducted different research on the construction and measurement methods of the Inclusive Financial Inclusion Development Index. Beck (2007) constructed the inclusive financial evaluation index system based on two dimensions of bank service penetration and usage by selecting eight indicators including the number of bank points per million square kilometers, the number of ATMs per million square kilometers and the proportion of GDP per capita[7]. Arora (2010) used relative indicators of financial accessibility to measure the level of inclusive financial development in developed and developing countries[8]. Mandira Sarma(2010) proposed IFI (Index of financial inclusion). This index absorbs important information from three dimensions of inclusive financial development. This index absorbs the important information of the three dimensions of inclusive financial development, such as financial institutions penetration, financial service availability and usage. It feels that these three dimensions are equally important and provides a much more comprehensive measurement method[9].Chakravarty(2010) improved the IFI index and calculated IFI based on the measurement of HDI, HPI, GDI, etc[10]. Gupte (2012) constructed the inclusive financial index from four dimensions: financial penetration, financial usage, financial transaction convenience, and financial transaction cost[11]. Rahman(2013) uses the weighted average of the four dimensions of financial penetration facilitation, financial use efficiency, financial absorption rate and satisfaction as the comprehensive inclusive finance index[12].

Chinese scholars' research on the inclusive financial evaluation index system is mostly based on the perspective of financial service providers, and has been improved in light of national conditions. Jiao J.P. (2015) built an evaluation system with 19 indicators in three dimensions and evaluated the level of inclusive finance in various provinces and cities in China firstly learning from the evaluation system of GPFI and joining the Chinese characteristics indicators such as agricultural support service points and personal credit file filing rate[13]. Du Q (2016) constructed an evaluation system with three dimensions and eight indicators combining three types of financial institutions, including banking, securities and insurance, and selected panel data of 31 provinces and cities to measure the development level of inclusive finance in various provinces and cities in China[14].

Scholars have studied farmer entrepreneurship from different angles. Papzan et al. (2008) pointed out that personality quality and management ability are important factors affecting farmers' entrepreneurship[15].Wolf (2007) proposes that personal skills, management skills, opportunity recognition ability, market risk prediction and control, crisis awareness, capital risk resolution, innovation ability, cooperation spirit and skills, etc are important quality of successful entrepreneurship from the perspective of farmers' entrepreneurial ability[16].Liu T.Y. (2010) believes that skills training, management ability, relatives and friends lending, financial lending, accessibility of natural resources, entrepreneurial motivation and attitudes toward risk are the main factors affecting farmers' entrepreneurial willingness[17]. Deng J.S. (2010)[18], Zhu H.G. (2013) believes that the financial environment, technical environment, social environment and institutional environment in the external environment have an impact on entrepreneurship[19].

Some scholars have also analyzed the relationship between inclusive finance and farmers from different perspectives. Hurst and Lusardi (2004) believe that high credit restraint and constraints are common in developing countries, and a relaxed credit environment can change the expected level of entrepreneurial farmers and increase risk appetite, therefore increase the tendency of farmers to start their own businesses[20]. Heino (2006) divided the borrowing methods into relatives and friends, loan companies, cooperative banks, agricultural banks, and postal savings, using cases of 3,329 “small rural entrepreneurial enterprises” confirmed that the main problem of rural entrepreneurship is the shortage of funds, and farmers have strong formal credit needs[21].Wang K.Y., Zhang H. W. (2016)makes an empirical analysis on the impact of rural inclusive development on rural households' entrepreneurship by using dynamic panel model and systematic GMM estimation method from 2005 to 2013[22].Li G. L. (2016) makes an empirical analysis of the level and influencing factors of inclusive finance development in 13 cities in Hei longjiang province[23].Wang X. H., et al(2016) selects the data from 2006 to 2010, analyzes the differences between financial inclusions in different provinces from different perspectives, and analyzes the influencing factors of rural financial inclusion development from an empirical perspective[24].Wang F. Y. and Tan M. J. (2017) analyzed the mechanism of the integration of inclusive finance and farmer entrepreneurship, the dilemma and causes of the two in the process of integration, and put forwards merge paths between the two from the macro level government, the meso-financial enterprise, and the micro-entrepreneurial farmer[25].

Chinese and other countries’ scholars have carried out more valuable research on the basic connotation of inclusive finance, the measurement of inclusive financial development, the entrepreneurship of farmers, and the relationship between inclusive finance and entrepreneurship. Although Inclusive Financial Inclusive Development Index is measured, there is a lack of trend analysis on the measured index. There is less empirical analysis on the impact of inclusive financial inclusive development on farmer entrepreneurship. Based on the existing research, this paper selects the latest data for analysis. At the same time, Kernel density non-parametric estimation method is used to analyze the trend of Inclusive Financial Inclusive Development Index, which can be used for reference by other scholars. When analyzing the impact of the Inclusive Financial Inclusion Development Index on farmer entrepreneurship, this paper uses static panel (Pooled-OLS, fixed-effect least squares estimation (FE-OLS), random effect least squares estimation (RE). -OLS)) and dynamic panel (differential GMM and system GMM) estimation methods for comparative analysis, selecting optimal system GMM estimation method, therefore the empirical analysis results can more deeply analyze the relationship between inclusive financial inclusive development level and farmer entrepreneurship, providing decision-making basis for the government to formulate targeted policies to promote farmer entrepreneurship.

## Theoretical analyses of IFI index and its impact on farmer entrepreneurship

### The inclusive finance inclusion development indicators

Mandira Sarma proposed the concept of the Financial Inclusion Development Index in 2010, followed by a large number of scholars choosing various indicators to construct the above index. Based on the previous studies and taking into account the availability of data, this paper selects indicators such as geographical permeability, utility and affordability. The details are detailed in Table 1.

**Table 1:** System of financial inclusion development index in China.

Index	Dimension	Measure index	Calculation method
**Financial inclusion development index**	**Geographical permeability**	Geographic dimension service permeability	Number of banking institutions / area
			Permeable banking institutions / area
		Population dimension service permeability	Number of banking institutions / Regional population
			Permeable banking institutions / Regional population
	**Utility**	Deposit	Financial institutions various deposit balances/GDP
		Loan	Financial institutions various loan balances/GDP
		Premium density	Premium income/Population
		Premium depth	Premium Earnings / GDP
	**Affordability**	Loan rate	Affordability rate upward floating loan ratio

Geographical permeability describes the extent of financial services coverage in the region and it is the basic level of financial inclusion, which reflects the difficulty of obtaining financial services. Research scholars at home and abroad basically use the number of banking institutions / regional area, the number of banking institutions / the number of population in the region. The reduction in the number of banking outlets does not represent a financial service. Sometimes to merger outlets but increase the number of employees can also provide more financial services to a certain extent. Therefore, the number of employees in the banking industry/area, the number of employees in the banking industry, and the number of people in the region are also taken into account. The higher the indicator, the higher the probability of obtaining financial services and the higher level of inclusive financial development.

Utility reflects the use of financial services, mainly expressing by deposits balance/GDP, loans balance/GDP, premium income balance/GDP and per capita premium income. Deposits, loans, and premiums reflect the use of financial services, and the weight of these indicators in GDP can reflect the utility of the use. The higher the indicator, the better the use of financial services, reflecting the higher level of financial inclusion development.

Affordability describes whether the cost of obtaining a financial service is within an affordable range. For most farmers, the financial burden is the cost of loan interest. The indicator of affordability is measured by the interest rate upward floating loan ratio. The higher the indicator, the higher the price of financial services and the lower the degree of financial inclusion development.

### Methodology for inclusive financial development index

The calculation of the comprehensive index of inclusive financial inclusion development is divided into three steps. Firstly, the coefficient of variation is used to determine how much weight each indicator occupies. The number of variances is the first thing we need to determine. We first calculate the average of the first indicator and its standard deviation (*s*_*i*_). *B*_*i*_ represents the coefficient of variation, which is calculated as follows:

$$B_{i}=\frac{s_{i}}{\bar{X_{i}}}$$ (1)

Then add the total sum and calculate the weight

$$w_{i}=\frac{B_{i}}{\sum_{i=1}^{n}B_{i}}$$ (2)

Secondly, the index values of each dimension are calculated. *X*_*i*_ represents the i dimension.

$$X_{i}=w_{i}*\frac{D_{i}-\min D_{i}}{\max D_{i}-\min D_{i}}$$ (3)

w_i_ is the weight calculated above, D_i_ is the true value of the i dimension. max*D*_*i*_ represents maximum value, and min*D*_*i*_ represents minimum value.

Thirdly, the comprehensive index value RFI of the inclusive financial inclusion development is measured according to the values and weights of each dimension.

$$IFI_{1}=\sqrt{\frac{\sum X_{i}^{2}}{\sum w_{i}^{2}}}$$ (4)

$$IFI_{2}=1-\frac{\sqrt{\sum {\left(w_{i}-X_{i}\right)}^{2}}}{\sqrt{\sum w_{i}^{2}}}$$ (5)

$$IFI=\frac{IFI_{1}+IFI_{2}}{2}$$ (6)

### The impact of inclusive IFI index on farmers' entrepreneurship

#### Model construction

This paper uses the dynamic panel model to analyze the impact of inclusive financial inclusion development on farmer entrepreneurship. STATA software is used for specific processing.

$$Y_{it}=\alpha Y_{it-1}+\beta X_{it}+u_{t}+\delta_{it}$$ (7)

where *Y*_*it*_ denotes the interpreted variable, *Y*_*it*−1_ is the first-order lag of the interpreted variable, *α* and *β* are the coefficients, *X*_*it*_ is the explanatory variable, *u*_*t*_ is the intercept term representing individual heterogeneity and it is the random interference term. The specific dynamic panel model constructed in this study is:

$$RH_{it}=\alpha_{0}RH_{it-1}+\alpha_{0}IFI_{it}+\beta X_{it}+u_{t}+\lambda_{it}$$ (8)

*RH* represents the measure of the rural entrepreneurial value of the explained variable. Because the explanatory variable of formula (8) contains the first-stage lag item of farmer entrepreneurship, a dynamic panel estimation method must be employed. Among them, the system GMM integrates the characteristics of the horizontal GMM and the differential GMM. Furthermore, the hysteresis of the differential variable is used as the instrumental variable of the horizontal equation, and the hysteresis of the horizontal variable is used as the instrumental variable of the differential equation. In this way, the system GMM estimation method not only improves the estimation efficiency by using more information, but it can also estimate the variables that do not change with time. Thus, the accuracy of the estimation result is higher than that of the differential GMM. To better compare the rationality of the empirical analysis results, this paper uses estimation methods such as static panel (Pooled_OLS), fixed effect least squares (FE-OLS), random effect least squares (RE-OLS), and dynamic panels (differential GMM and system GMM).

#### The variables

IFI is the inclusive financial inclusion development index, the results measured above indicate that the larger the indicator, the higher the level of inclusive financial inclusion development in the region.

*X* represents the control variable, *i* represents the control variable, *t* represents the time, *α* and *β* represent the coefficients to be estimated. The control variables in this study mainly include urbanization level (CZHH), investment in fixed assets (GDZT), farmer income level (LNNSH), economic openness (OPEN), education level (EDU), and economic development level (GDP).

The level of urbanization (CZHH) is expressed by the ratio of urban population to total population in each province. With the acceleration of urbanization, it has provided more opportunities and choices for farmers to start their own businesses, which has greatly promoted farmers to start non-farm businesses. At present, however, the true rate of urbanization is only 35%. More and more people are facing high costs in big cities and will choose to go to rural areas to start their own businesses. At the same time, many 40-year-old and 50-year-old migrant workers have no sense of belonging in cities. "Some of the post-1980s and post-1990s migrant workers face real needs and will return to rural areas to start businesses and participate in the construction of new rural areas. The impact of coefficient on farmers' entrepreneurship is uncertain. The data comes from the Chinese statistical yearbook.

The impact of farmer's fixed asset investment (GDZT) on farmer entrepreneurship is uncertain. Farmers' fixed asset investment will be invested in some infrastructures that are conducive to entrepreneurship, which will help improve the efficiency of farmers' operations and at the same time play a leading role in demonstration. If the farmer's fixed asset investment is invested in real estate such as a house, the funds used for starting the business will be reduced, which will affect the entrepreneurship of the farmer to a certain extent. Its coefficient of influence on farmer entrepreneurship is uncertain. To maintain the stability of the data, the logarithm is processed in the empirical analysis and is represented by LNGDZT.

The income level of farmers (LNNSH) is derived from the income level of urban and rural residents(farmers) in various regions in the "China Rural Statistics Yearbook". In the survey of 53 entrepreneurial households in C village, it can be seen that most of the funds needed by farmers to start a business in the early stages come from their own accumulation in the early stages. The income level of farmers is the basis for farmers to start a business and directly relates to the willingness of farmers to start a business, its specific impact on farmer entrepreneurship is uncertain. To maintain the stability of the data, the logarithm is processed in the empirical analysis and is represented by LNNSH.

The indicator economic openness (OPEN) is used in the usual formula to calculate the total import and export ratio of GDP. The data is obtained from the total import and export of goods in the China Statistical Yearbook. The more open the economy, the more developed the finance, and the more conducive the index is to rural entrepreneurship.

Under normal circumstances, the degree of education (EDU) has a positive impact on the entrepreneurship of farmers. At the same time, the higher the education level of the farmer, the more employment opportunities will affect the enthusiasm of entrepreneurship. The farmers with low education levels experience a follow-up phenomenon whereby they see what others do to make money and tend to do the same. In reality, there is uncertainty about the impact of education on farmer entrepreneurship. The degree of education expressed by the data indicate the proportion of those with high school and middle school educations, those with a secondary school education, and the sum of junior college and college degrees. The data are obtained from the Rural Statistics Yearbook of China.

There is a positive correlation between the level of economic development and the entrepreneurship of farmers with the two have mutually reinforcing roles. The per capita GDP is used to indicate the level of economic development. The data are obtained from the China Statistical Yearbook.

## Empirical analyses

### Data

This study obtains data for the period 2004 to 2016 from four municipalities and 22 provinces, and then, based on the obtained data, it forecasts the data for 2017. The number of banking institutions and the number of bank employees referenced in the article are derived from the annual financial operation report of each province. Data on deposits, loans, regional population, and regional GDP are derived from the Statistical Bulletin of National Economic and Social Development for each year and from the WIND database. In this paper, we use the sum of the number of rural private enterprise investors and the number of rural individual employed as the proportion of the entire rural population to indicate the measure of rural entrepreneurship (RH). The data come from the number of private enterprises employed in the "China Statistical Yearbook"(rural areas), the number of individuals employed in the sub-region (rural areas), and the composition of the urban and rural population in the sub-region[26].

A 2016 survey of 53 entrepreneurial households in village C of Suqian City, China found that 30 of the 53 entrepreneurial households demonstrated credit needs, while only 19 received loans from formal financial institutions and of these 19, most were provided by rural commercial banks[27]. It is further acknowledged that farmers are affected by financial development during the process of starting a business. Farmers’ entrepreneurship, however, is also affected by other factors, such as their income, level of urbanization, level of education, the openness of regional economies, and large macroeconomic policies. The data comes from the "China Statistical Yearbook" and "China Rural Statistical Yearbook." The descriptive statistical analysis of each variable is detailed in Table 2.

**Table 2:** Descriptive statistical analysis of variables.

Variable	Mean	Median	Maximum	Minimum	Std. Dev.
**RH**	18.56	7.83	195.32	1.62	31.70
**RFI**	0.23	0.20	0.77	0.04	0.12
**CZHH**	53.35	50.75	89.60	26.87	15.37
**LNGDZT**	5.11	5.45	7.01	0.41	1.26
**LNNSH**	8.80	8.85	10.16	7.45	0.61
**OPEN**	0.36	0.14	1.80	0.03	0.44
**EDU**	16.93	16.90	49.78	5.90	7.66
**GDP**	3.76	3.24	11.83	0.59	2.51

As evidenced from Table 2, the standard deviations of the three indicators of the inclusive financial inclusion development index as well as the standard deviations of economic openness and the regional economic development level are not excessively large. There is a substantial difference in the standard of indicators such as rural household entrepreneurship, urbanization level, fixed asset investment, farmer income, and education level mainly because of the large differences between and among the regions themselves.

### Results of the inclusive financial inclusion development index

**Fig 1:**
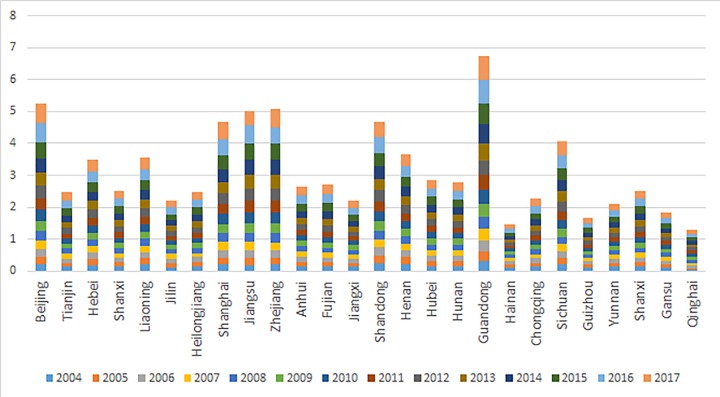
Comparison of inclusive financial inclusion development indices of various regions.

**Fig 2:**
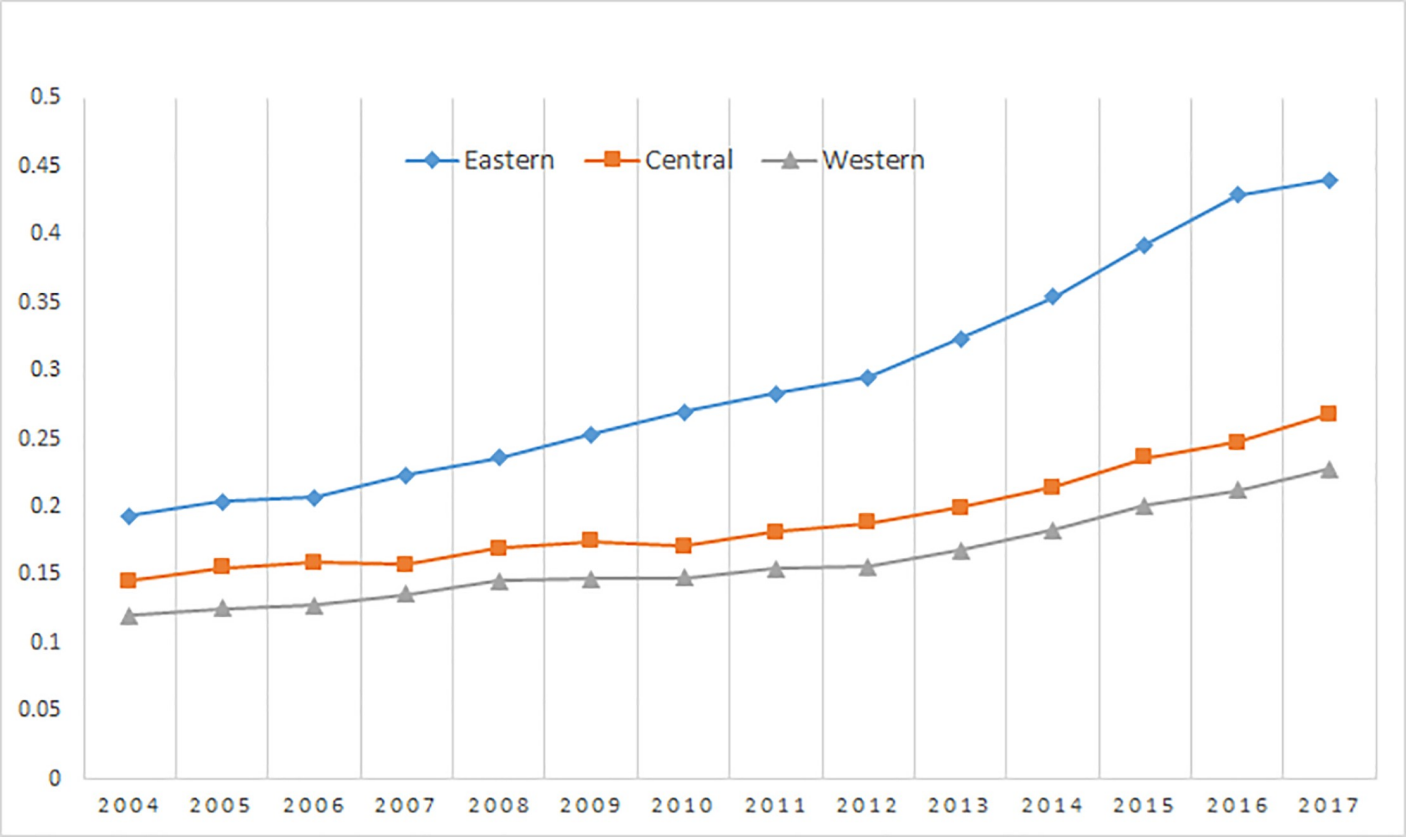
Comparison of inclusive financial inclusion development index in various regions.

Using the above formula, the inclusive financial inclusion development index of four municipalities and 22 provinces from 2004 to 2017 is calculated. The details are presented in Table 3. The higher the inclusive financial inclusion development index, the higher the financial inclusion in the region. This study claims that a financial inclusion development index below 0.2 is considered to be low; an index score between 0.2 and 0.35 is considered to be medium; an index score between 0.36 and 0.5 is considered to be high medium, and an index score greater than 0.5 is considered to be high. There are certain gaps in the inclusive financial inclusion development index in different regions, as presented in (Fig 1).The degree of inclusive development and the ranking of inclusive finance are based on the results of the 2017 calculations. While there is a gap in the economic development of the eastern, central and western regions of China, there is also a clear gap in the inclusive financial inclusion development index, that is the index of the eastern region>that o the central region>that of the western region, as presented in (Fig 2). Nonetheless, the gap between the inclusive financial inclusion development indices of various regions of China is not excessively large in any given year. Eastern: Beijing, Tianjin, Hebei, Liaoning, Shanghai, Jiangsu, Zhejiang, Fujian, Shandong, Guangdong, Hainan. Central: Shanxi, Jilin, Heilongjiang, Anhui, Jiangxi, Henan, Hubei, Hunan. Western:Chongqing, Sichuan, Guizhou, Yunnan, Shanxi, Gansu, Qinghai.

**Table 3:** Financial inclusion development indices in various regions of China.

Region	2004	2005	2006	2007	2008	2009	2010	2011	2012	2013	2014	2015	2016	2017	degree	Ranking
**Beijing**	0.21	0.25	0.24	0.26	0.28	0.32	0.36	0.36	0.38	0.41	0.45	0.52	0.58	0.63	high	2
**Tianjin**	0.14	0.12	0.14	0.14	0.16	0.16	0.15	0.16	0.17	0.18	0.20	0.23	0.25	0.26	medium	13
**Hebei**	0.19	0.19	0.19	0.20	0.21	0.23	0.23	0.24	0.25	0.26	0.28	0.31	0.34	0.36	higher	8
**Shanxi**	0.13	0.13	0.14	0.13	0.16	0.17	0.16	0.18	0.19	0.20	0.20	0.22	0.24	0.25	medium	14
**Liaoning**	0.22	0.19	0.18	0.20	0.22	0.22	0.23	0.25	0.25	0.28	0.30	0.33	0.34	0.36	higher	8
**Jilin**	0.12	0.13	0.15	0.14	0.14	0.14	0.15	0.14	0.16	0.15	0.17	0.19	0.21	0.22	medium	16
**Heilongjiang**	0.15	0.15	0.14	0.13	0.16	0.16	0.16	0.16	0.17	0.19	0.21	0.22	0.23	0.25	medium	14
**Shanghai**	0.20	0.21	0.24	0.26	0.27	0.29	0.31	0.31	0.33	0.35	0.39	0.45	0.50	0.53	high	4
**Jiangsu**	0.20	0.23	0.23	0.25	0.28	0.31	0.34	0.36	0.40	0.43	0.47	0.52	0.58	0.44	higher	6
**Zhejiang**	0.20	0.22	0.23	0.26	0.28	0.31	0.34	0.37	0.39	0.43	0.49	0.49	0.53	0.57	high	3
**Anhui**	0.14	0.15	0.16	0.16	0.16	0.17	0.17	0.18	0.18	0.20	0.21	0.24	0.25	0.27	medium	12
**Fujian**	0.14	0.14	0.14	0.15	0.16	0.15	0.16	0.18	0.20	0.22	0.24	0.26	0.28	0.30	medium	9
**Jiangxi**	0.13	0.12	0.13	0.14	0.14	0.14	0.14	0.15	0.15	0.16	0.17	0.19	0.21	0.22	medium	16
**Shandong**	0.23	0.25	0.26	0.26	0.26	0.29	0.31	0.33	0.35	0.36	0.40	0.42	0.48	0.50	high	5
**Henan**	0.22	0.22	0.21	0.21	0.23	0.23	0.23	0.25	0.26	0.28	0.29	0.32	0.34	0.36	higher	8
**Hubei**	0.13	0.17	0.17	0.17	0.19	0.19	0.19	0.20	0.21	0.22	0.24	0.26	0.23	0.29	medium	10
**Hunan**	0.15	0.16	0.17	0.17	0.17	0.19	0.18	0.19	0.20	0.20	0.22	0.24	0.26	0.28	medium	11
**Guandong**	0.32	0.32	0.33	0.36	0.38	0.41	0.44	0.47	0.44	0.54	0.60	0.66	0.72	0.77	high	1
**Hainan**	0.10	0.11	0.10	0.12	0.09	0.10	0.09	0.09	0.09	0.10	0.10	0.12	0.13	0.13	lower	21
**Chongqing**	0.13	0.13	0.14	0.13	0.14	0.15	0.14	0.15	0.15	0.16	0.18	0.20	0.21	0.24	medium	15
**Sichuan**	0.20	0.21	0.21	0.22	0.23	0.24	0.28	0.29	0.30	0.32	0.35	0.37	0.40	0.43	higher	7
**Guizhou**	0.10	0.10	0.10	0.11	0.11	0.11	0.10	0.11	0.11	0.12	0.13	0.15	0.16	0.17	lower	19
**Yunnan**	0.13	0.13	0.12	0.13	0.14	0.15	0.13	0.14	0.14	0.15	0.17	0.18	0.19	0.20	medium	17
**Shanxi**	0.14	0.15	0.14	0.15	0.16	0.16	0.17	0.17	0.17	0.19	0.21	0.23	0.24	0.26	medium	13
**Gansu**	0.10	0.11	0.11	0.12	0.13	0.12	0.12	0.13	0.12	0.14	0.14	0.16	0.17	0.18	lower	18
**Qinghai**	0.04	0.06	0.07	0.09	0.11	0.09	0.09	0.10	0.09	0.09	0.11	0.12	0.11	0.13	lower	20

### Results of empirical analysis of Kernel density estimation

Kernel density estimation is a non-parametric estimation method that can analyze the trend of the distribution pattern of the research object with respect to time. The higher the kurtosis, the denser the data are, and the larger the width, the wider the data distribution and the smoother the curve. This paper uses the STATA software to estimate the kernel density of the evolution of the inclusive financial inclusion development index for the whole country as well as Western central and western regions.

**Fig 3:**
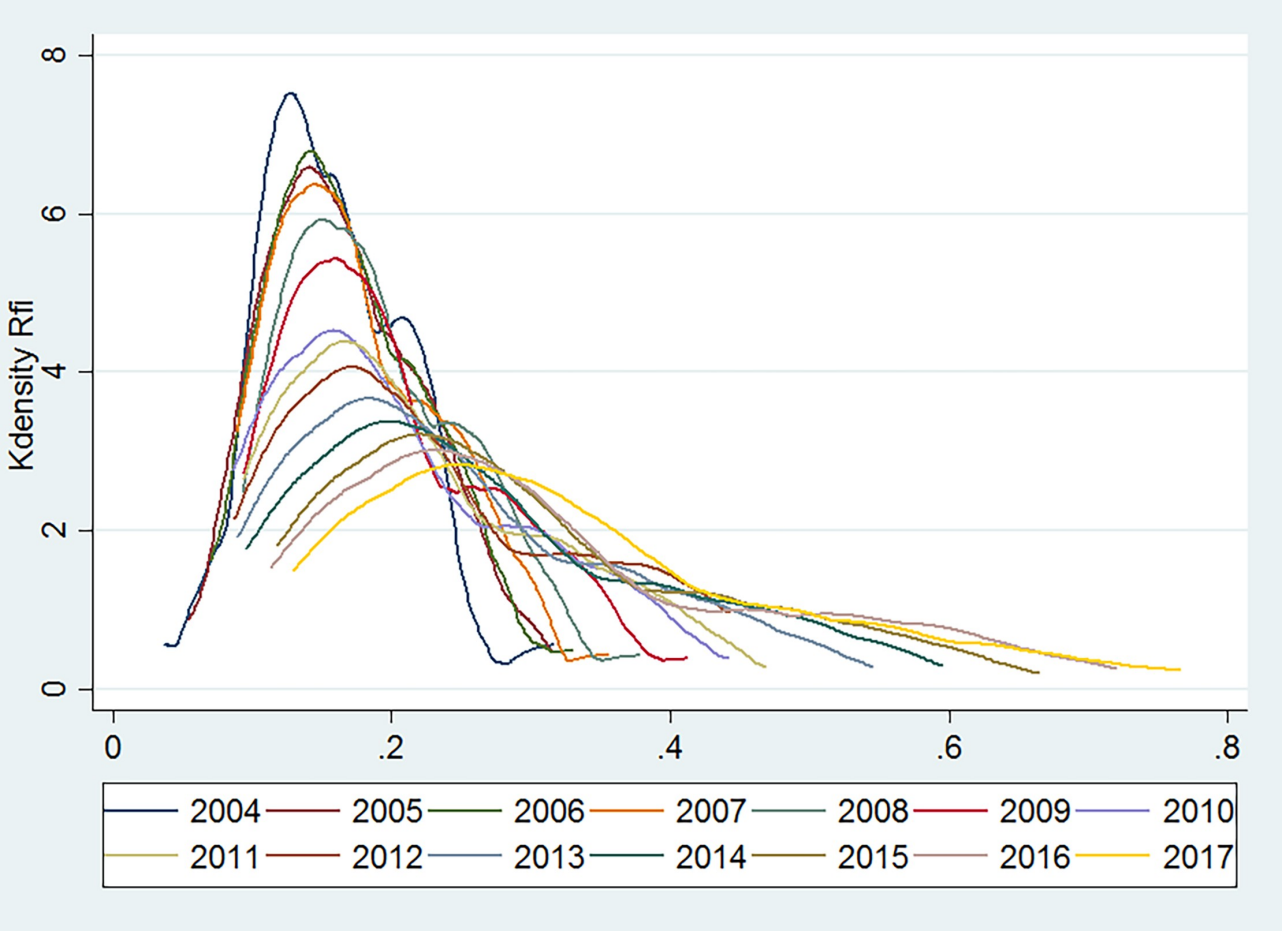
Development of inclusive finance in China.

Fig 3 presents the trend of the inclusive financial inclusion development index relative to 26 provinces and four municipalities across the country. The peaks and the width tend to be flat. The inclusive financial development level of all regions reveals an overall upward trend. Before 2010, these regional differences are not prominent, whereas the trend of differentiation among the various regions after 2010 is obvious.

**Fig 4:**
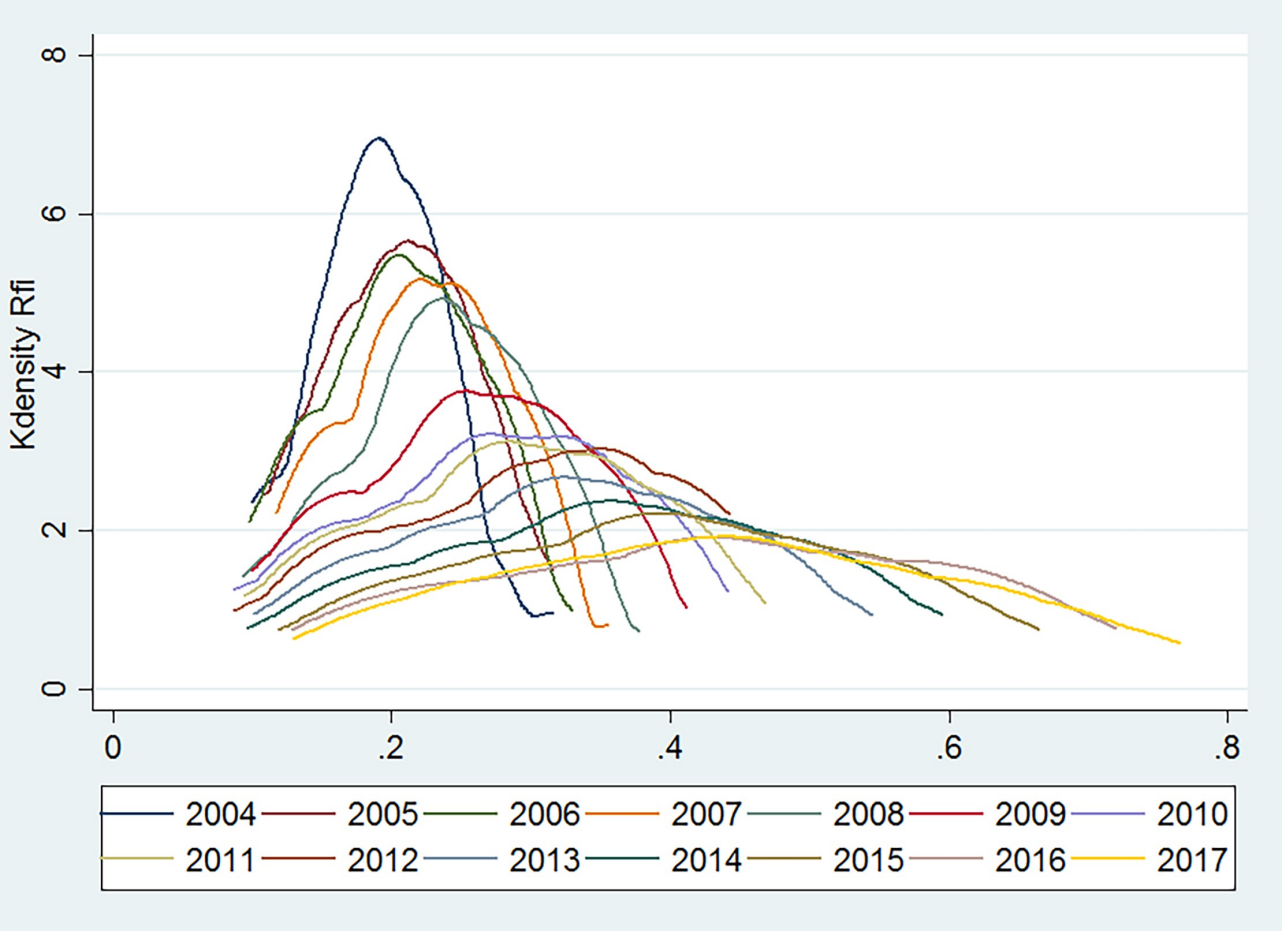
The development of inclusive finance in the eastern region.

Fig 4 presents the inclusive financial development of the eastern region. Between 2004 and 2008, the peaks were steep and narrow, and the level of inclusive financial inclusion in the eastern region was close. After 2009, the peaks gradually became steeper and the trending gradually closed to the normal distribution. However, the overall level of inclusive financial inclusive development in all regions increased rapidly from 2004 to 2017, and the gap between the inclusive financial inclusive development levels in various regions continued to increase.

**Fig 5:**
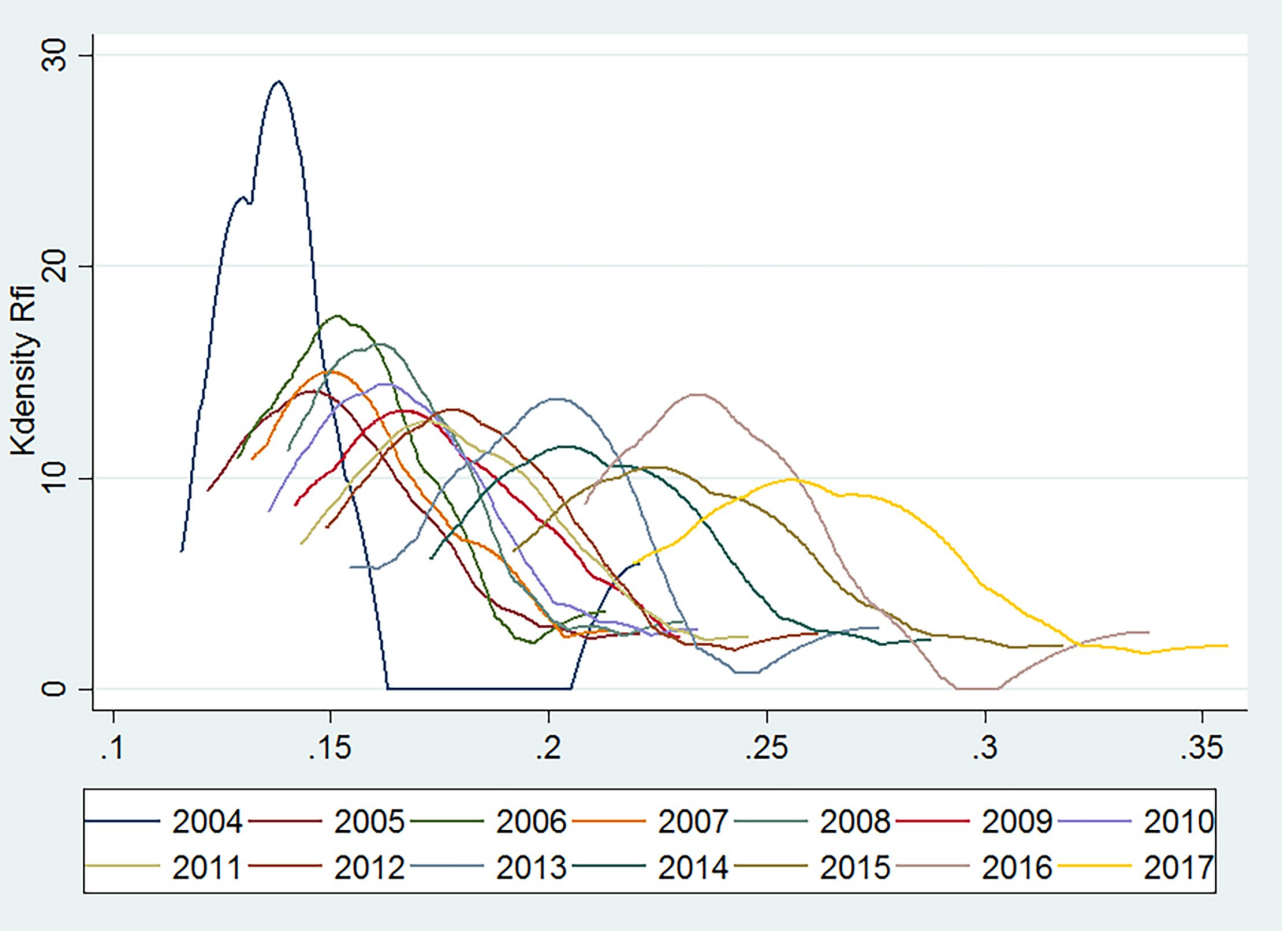
The development of inclusive finance in the central region.

Fig 5 presents the development of inclusive financial inclusion in the central region. In 2004, the overall level of inclusive financial inclusion development in each region was relatively close. Of the regions, the level of Henan Province was higher than that of other provinces, causing the graph to appear steeper and gentler. In other years, the level of inclusive financial inclusion development in the central regions exhibited an increasing trend, and accordingly, the differences between the regions increased. However, the overall level was not very high and was, in fact, lower than that of the eastern region.

**Fig 6:**
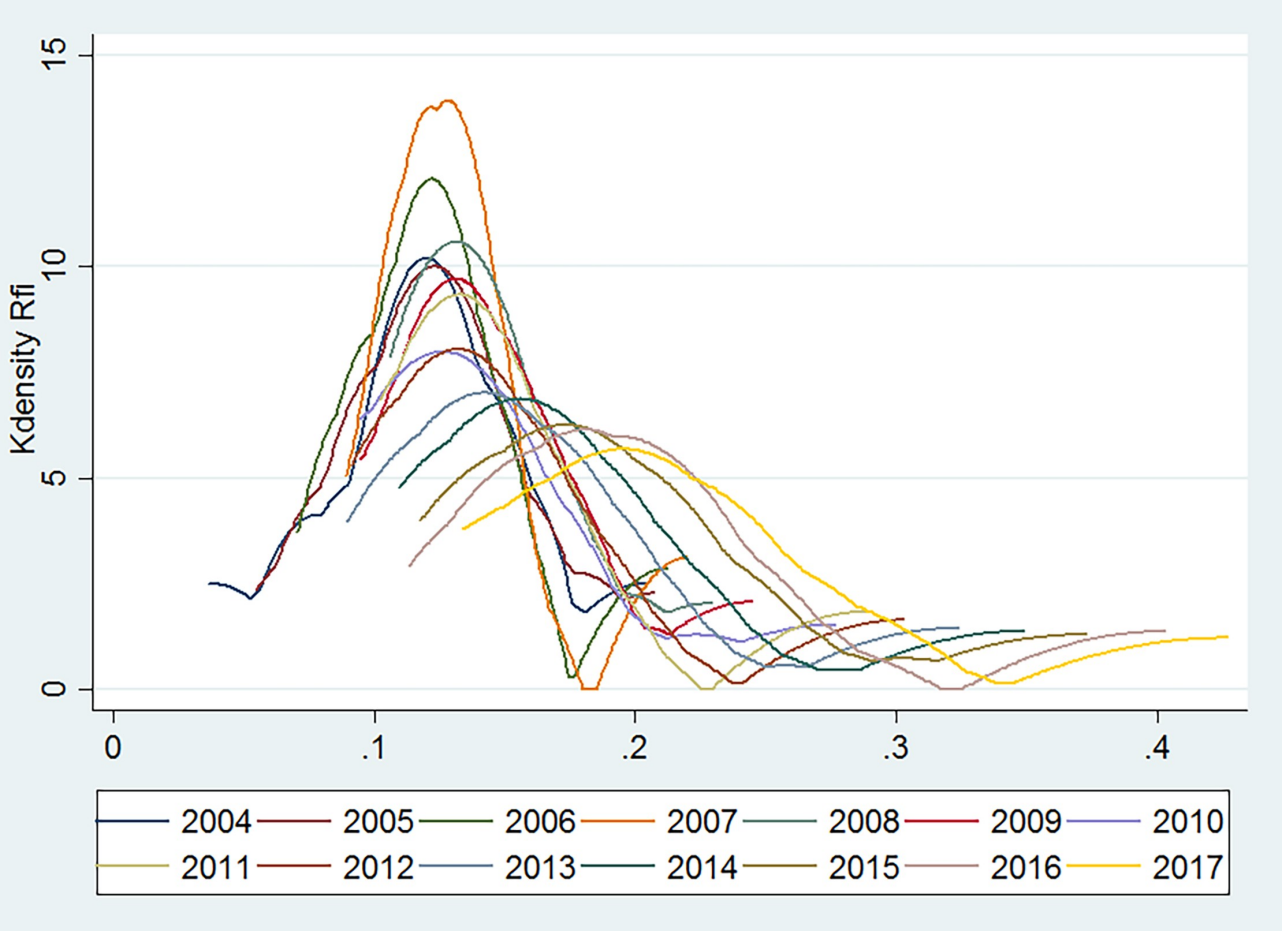
The development of inclusive finance in the western region.

Fig 6 displays the development of inclusive financial inclusion in the western region. The overall level of inclusive financial inclusion development in all regions is lower than the levels in the eastern and central regions. The trend of the graphs rises first, then decreases and then slightly increases with an overall right shift. These data indicate that the overall trend of inclusive financial inclusion in various areas of the western region is on the rise, albeit fluctuations do exist.

### Results of empirical analysis on the impact of inclusive financial development index on farmer entrepreneurship

The impact of inclusive financial development index on farmer entrepreneurship is detailed in Table 4.

**Table 4:** Estimation of the impact of inclusive financial inclusion development on farmer entrepreneurship.

	Static Panel			Dynamic Panel	
	OLS	FE	RE	DIF-GMM	SYS-GMM
**RH(-1)**				-0.191* (-1.66)	-0.231*** (-4.55)
**RFI**	129.839*** (7.88)	90.536*** (6.82)	87.359*** (6.71)	237.076*** (3.36)	148.309*** (10.61)
**CZHH**	-0.673*** (-3.21)	-1.105*** (-3.87)	-0.548** (-2.11)	-0.235 (-0.36)	3.878*** (18.81)
**LNNSH**	25.436*** (5.32)	3.236 (0.75)	1.224 (0.29)	-25.953*** (-3.20)	-28.442** (-2.34)
**LNGDZT**	-17.348*** (-13.72)	2.358 (1.32)	-4.500*** (-2.87)	1.795 (1.13)	0.603 (0.65)
**EDU**	0.603*** (3.44)	-0.214 (-1.11)	0.029 (0.15)	-0.761 (-1.63)	-4.146*** (-19.25)
**OPEN**	9.611** (1.99)	8.392 (1.57)	16.677*** (3.41)	7.239 (0.64)	13.271*** (4.57)
**GDP**	-1.809 (-1.33)	2.784*** (3.29)	3.673*** (4.22)	6.277*** (4.05)	8.795*** (5.37)
**_cons**	-117.003*** (-3.47)	7.240 (0.31)	19.369 (0.83)		70.753 (0.66)
***N***	275	275	275	165	227
**adj. *R***^**2**^	0.764	0.479			
**F**	127.838	40.613			
**White test**	Heteroscedasticity				
	274.62***				
**BG test**	autocorrelation				
	2.937*				
**Wald test**		Inter-group heteroscedasticity	Inter-group heteroscedasticity		
		5337.65***	5337.65***		
**Woolwich Ridge F Test**		Group autocorrelation	Group autocorrelation		
		54.436***	54.436***		
**Pesaran's test**		Concurrent correlation between groups	Concurrent correlation between groups		
**Arellano-Bond**				0.694	0.193
**Hansen test**				0.193	0.659

t statistics in parentheses

* p < .1

** p<0.05

*** p<0.01.

The heteroscedasticity and sequence correlation test on the model found that the heteroscedasticity and autocorrelation problems exist in the mixed OLS regression, and the heteroscedasticity needs to be processed. The heteroscedasticity and autocorrelation problems exist in the fixed-effect regression and random-effect regression models, and the heteroscedasticity needs to be Autocorrelation is processed. Considering that the system generalized moment estimation method (SYS-GMM) can ignore the sequence correlation and heteroscedasticity of data existence, it is not necessary to understand the distribution information of random error terms. While considering the difference generalized moment estimation method (DIF-GMM) the system generalized moment estimation method (SYS-GMM) has advantages in analyzing dynamic panel data. Therefore, the paper finally uses the systematic generalized moment estimation method for empirical analysis, mixed OLS, fixed-effect regression, random effect regression and differential generalized moment estimation are used as references.

After the residual test, the Hansen test and Arellano-Bond's p value in the GMM estimate were both greater than 0.1. Therefore, the original assumption that "all tool variables are valid" and "all disturbance terms are not self-correlated" cannot be rejected at the 10% significance level, that is, the model passes the over-recognition test and the sequence correlation test. Therefore, the estimated results of the system GMM are valid.

The inclusive financial inclusion development index is significant among the estimation methods, and the front coefficient is positive, suggesting that the higher the degree of inclusion development of inclusive finance, the more conducive the index is to farmer entrepreneurship. Furthermore, the better the development of inclusive finance in all regions, the easier it is for farmers to obtain financial support from all sides, a situation that can greatly promote the enthusiasm of farmers to start their own businesses and solve the problem of funding difficulties as farmer seek to become entrepreneurs. The level of urbanization (CZHH) is significant in the system GMM estimation method. The former coefficient is positive, and the higher the level of urbanization the more favorable to farmers' entrepreneurship through promoting market development and broadening the entrepreneurial channels of farmers, especially the development of non-agricultural industries. The income level of farmers is estimated to be significant in the systematic GMM estimation method. The former coefficient is negative. When the farmers' income level is high, the entrepreneurial enthusiasm is weakened to a certain extent. The degree of education is estimated to be significant in the systematic GMM estimation method. The former coefficient is negative, mainly because the employment of this sector group with higher education level is easier, therefore they are not inclined to start a business. The degree of economic openness and regional economic development are estimated to be significant in the systematic GMM estimation method, and the front coefficient is positive. The higher the degree of economic openness and the level of regional economic development, the more favorable it is for farmers to start businesses.

### Conclusions

Through calculations, it is found that the gap between the inclusive financial inclusive development index in different regions of China is relatively large. In general, the inclusive financial inclusion development in the eastern region with better economic development is higher, and the inclusive financial inclusion development in the western region with weaker economic development is lower. This paper uses dynamic panel estimation method to make an empirical analysis of the impact of inclusive financial inclusion development on farmer entrepreneurship based the calculation of the inclusive financial inclusion development index of each province in China as a very important explanatory variable. In the empirical analysis, it is found that the inclusive financial inclusion index has a very significant positive impact on the entrepreneurship of the farmer, which is consistent with the government's policy orientation to promote the entrepreneurial entrepreneurship by developing the inclusive financial inclusive development level. The level of urbanization, economic openness, and regional economic development level have a significant positive impact on the entrepreneurship of farmers, which is consistent with the government's goal of creating a good economic environment and promoting the entrepreneurship of farmers. The income level of farmers and the level of education have a significant negative impact on the entrepreneurship of farmers. This is consistent with the actual situation of entrepreneurial farmers in China. The lower the income level and the lower the level of education, the higher the enthusiasm for entrepreneurship.

## Countermeasures

Based on the calculation that the inclusive financial inclusion development index of all provinces in China is an important interpretation variable, this paper uses dynamic panel estimation methods to conduct an empirical analysis of the impact of inclusive financial inclusion development on farmer entrepreneurship. The results of the empirical analysis indicate that the inclusive financial inclusion development index, urbanization level, economic openness, education level, and economic development level have a significant impact on farmer entrepreneurship. Specific recommendations are as follows:

### Improving inclusive finance inclusion development to better promote farmer entrepreneurship

In a survey of entrepreneurial farmers in C village and a 2016 survey of 306 family farmers entrepreneurial processes in Suqian City, it was determined that credit constraints exist with respect to farmer entrepreneurship and family farmer entrepreneurship and that the level of inclusive development of inclusive finance directly restricts farmers entrepreneurship [28]. To better promote rural households to start their own businesses, the level of inclusive financial development should constantly be raised. The calculations of the inclusive financial inclusion development index of the provinces indicate that some provinces have lower levels of inclusive financial development and have greater room for improvement. T strengthen the support of policy-oriented financial institutions towards farmer entrepreneurship and better solve the problem of credit constraints regarding farmers entrepreneurship, it is necessary to create innovative loan forms, reduce loan costs and reduce loan procedures. Furthermore, to as great a degree as possible, the top-level design of inclusive financial inclusion development should strengthened and guided by the level of demand, and the environment should be driven to create more financial service platforms that benefit the people. At the same time, along with the development of Internet finance, the inclusive development of inclusive finance can make full use of the Internet, reduce the cost of financial services, and effectively promote the overall development of inclusive finance.

### Combine with urbanization and better support farmers' entrepreneurship

The rapid development of urbanization is conducive to promoting urban and rural development and prosperity. To better support farmers’ entrepreneurship, on the one hand, we can coordinate urban and rural development, institutionally protect farmers' entrepreneurship, establish good entrepreneurial mechanisms for land-losing farmers and encouraging farmers to actively explore various types of entrepreneurial forms; on the other hand, we can use implementation of policies such as handle licenses, credit support, water supply, and rent reduction and children's enrollment to reduce the threshold for farmers' entrepreneurship and better support farmers' entrepreneurship.

### Government increases investments in rural productive assets and better supports farmer entrepreneurship

Farmers' funds are limited. If there are more fixed assets investments, the funds that can be used for starting a business will be less, and entrepreneurship will be affected. The more productive the fixed assets investments are, the more conducive it is for farmers to start businesses. At present, housing investments account for a relatively large proportion of farmers’ fixed assets investments, whereas large-scale machinery, equipment and infrastructure investments account for a relatively small proportion. To better support farmer entrepreneurship, the government can increase financial inputs and increase investments in rural productive assets.

### Improve economic openness, improve regional economic development level and better support farmer entrepreneurship

Economic openness has a positive impact on farmer entrepreneurship, and it can provide a broader space for farmers to start businesses. Furthermore, we can promote the implementation of the going out, introducing strategy and promote the internationalization of agricultural products, which will provide farmers with a strong entrepreneurial environment. With the understanding that higher levels of regional economic development result in more entrepreneurial opportunities for farmers, different development measures in combination with regional advantages can be implemented to improve the level of regional economic development and better promote the entrepreneurship of farmers.

In the calculation of the Inclusive Financial Inclusion Development Index, this paper represents the banking industry and insurance industry indicators, but does not include the securities industry indicators. With the development of financial markets and the awareness of public investment, it is necessary to take the securities industry indicators into consideration in the future, therefore the inclusive financial inclusion development index is more comprehensive. In addition, with the development of digital finance, some services provided by bank outlets and practitioners are weakening. Some services can be obtained through mobile banking, e-banking, WeChat, Alipay and other channels. In the future, when evaluating the Inclusive Financial Inclusion Development Index, digital finance should also be taken into consideration.

## Supporting information

S1 File Inclusive financial inclusion development data. (XLSX)

S2 File Paneldata. (XLSX)
